# Detection and Surveillance of Invasive *Aedes* Mosquitoes in Spain: Implications for Arbovirus Risk and Public Health Preparedness

**DOI:** 10.3390/insects17090914

**Published:** 2026-09-01

**Authors:** Marta Bononad Brugger, M. Adela Valero, Agustín Llopis-Gonzalez, Maria Morales-Suárez-Varela

**Affiliations:** 1 Research Group in Social and Nutritional Epidemiology, Pharmacoepidemiology and Public Health, Department of Preventive Medicine and Public Health, Food Sciences, Toxicology and Forensic Medicine, Faculty of Pharmacy and Food Sciences, Universitat de València, 46100 Valencia, Spain; bobrug@alumni.uv.es (M.B.B.); agustin.llopis@uv.es (A.L.-G.); 2 Department of Pharmacy and Pharmaceutical Technology and Parasitology, Faculty of Pharmacy and Food Sciences, Universitat de Valencia, 46100 Valencia, Spain; madela.valero@uv.es; 3 Biomedical Research Centre in Infectious Disease (CIBERINFEC), Carlos III Health Institute, 28029 Madrid, Spain; 4 Biomedical Research Centre in Epidemiology and Public Health Network (CIBERESP), Carlos III Health Institute, 28029 Madrid, Spain

**Keywords:** *Aedes* mosquitoes, vector surveillance, arboviruses, Spain, public health

## Abstract

The expansion of invasive mosquito species of the genus *Aedes* has become a growing public health concern in Europe due to their role in the transmission of arboviruses such as dengue, Zika, and chikungunya. Early detection and surveillance of these vectors are essential to prevent the establishment of local transmission cycles. This study presents a review of surveillance reports and rapid risk assessments issued by the Health Alerts and Emergencies Coordination Centre of the Spanish Ministry of Health, complemented by relevant scientific literature on the presence and spread of *Aedes albopictus*, *Aedes aegypti*, and *Aedes japonicus* in Spain. The analysis describes the evolution of vector detection between 2004 and 2026, identifies geographical patterns of expansion, and examines the surveillance and response mechanisms implemented by Spanish health authorities. Particular attention is given to environmental, climatic, and socioeconomic factors that may facilitate the establishment of these invasive species. The results highlight the importance of strengthening entomological surveillance systems, improving coordination between national and regional health authorities, and enhancing early warning mechanisms to reduce the risk of arbovirus transmission in Spain. Unlike previous European reviews, this study integrates surveillance reports, grey literature, citizen science contributions, and scientific evidence to provide a comprehensive assessment of invasive *Aedes* surveillance in Spain and identify priority actions for strengthening arbovirus preparedness.

## Simple Summary

Invasive mosquitoes are spreading across Europe and have become an increasing public health concern because they can transmit diseases such as dengue, chikungunya, and Zika. Spain has experienced a rapid expansion of these mosquitoes during the last two decades, increasing the need for effective surveillance and early detection. This review summarizes the current knowledge on the distribution of invasive *Aedes* mosquitoes in Spain and examines the surveillance systems used to monitor their spread. It also discusses the environmental and human factors that contribute to mosquito expansion, including climate change, international travel, trade, and urbanization. In addition, the review compares surveillance strategies implemented in Spain with those used in other Mediterranean countries and highlights the growing contribution of citizen participation through digital reporting platforms. Understanding how these mosquitoes spread and how they are monitored can help improve preparedness and support timely public health responses. The findings of this review may assist health authorities, researchers, and policy makers in strengthening surveillance programs and reducing the risk of future outbreaks of mosquito-borne diseases.

## 1. Introduction

Vector-borne diseases represent an increasing public health concern worldwide, particularly due to the expansion of invasive mosquito species capable of transmitting arboviruses that affect human populations. Among these vectors, species belonging to the genus *Aedes* are of particular epidemiological relevance because of their ability to transmit pathogens such as dengue virus (DENV), chikungunya virus (CHIKV), Zika virus (ZIKV), and yellow fever virus (YFV). The global distribution of these arboviruses has expanded considerably in recent decades, largely driven by environmental changes, globalization, and increased international mobility [1,2].

The mosquito species *Aedes albopictus*, commonly known as the Asian tiger mosquito, is considered one of the most invasive mosquito species worldwide. Originally native to Southeast Asia, *Ae. albopictus* has spread rapidly to multiple continents over the last four decades. This expansion has been facilitated mainly through global trade and transportation networks, particularly through the international trade of used tyres and ornamental plants such as *Dracaena sanderiana* [3,4]. In addition, passive dispersal through road transportation has been identified as an important mechanism contributing to the rapid spread of *Ae. albopictus* across newly invaded regions [5]. Once introduced, the species has demonstrated a remarkable capacity to adapt to different climatic conditions and urban environments, allowing it to colonize temperate regions that were previously considered unsuitable for tropical mosquito species [6,7,8].

Beyond its rapid geographical expansion, *Aedes albopictus* has demonstrated remarkable ecological plasticity and adaptation to a wide range of environmental conditions. Recent evidence indicates that the species is capable of exploiting diverse vertebrate hosts and maintaining arboviruses through both horizontal and vertical transmission pathways. These characteristics contribute to its epidemiological relevance and may facilitate the persistence of arboviruses during inter-epidemic periods, increasing the risk of future outbreaks in newly colonized areas [9].

In Europe, the presence of invasive *Aedes* mosquitoes has increased substantially since the late twentieth century. *Ae. albopictus* was first detected in Europe in Albania in 1979 and subsequently expanded to Italy in the early 1990s. Since then, the species has progressively spread throughout southern and central Europe, including France, Spain, Greece, and several Balkan countries [10,11,12]. The establishment of this vector in multiple European regions has raised concerns about the potential emergence of local transmission of arboviruses in areas where these diseases were previously absent [13].

In addition to *Aedes albopictus*, Europe is experiencing the continued expansion of other invasive mosquito species of public health concern, including *Aedes aegypti*, *Aedes japonicus*, and *Aedes koreicus.* The re-establishment and spread of these species in several European countries have increased concerns regarding the potential emergence of dengue, chikungunya, and Zika virus transmission. Recent evidence indicates that southern Europe is becoming increasingly suitable for the establishment of invasive mosquito populations due to the combined effects of globalization, environmental change, and increasing human mobility [14].

Spain represents a particularly relevant geographical area for the surveillance of invasive mosquito species. The country combines climatic conditions favourable to mosquito survival with intense international tourism, extensive trade networks, and large urban coastal areas. These factors create suitable ecological conditions for the introduction and establishment of invasive mosquito populations. The first detection of *Ae. albopictus* in Spain was reported in Catalonia in 2004, and since then the species has expanded progressively along the Mediterranean coast and into several inland territories [15,16].

In addition to *Ae. albopictus*, other invasive mosquito species of the genus *Aedes* have been detected in Europe, including *Aedes aegypti* and *Aedes japonicus*. *Ae. aegypti*, the primary vector of dengue worldwide, has been detected sporadically in the Canary Islands, although stable populations have not been confirmed in mainland Spain. The Canary Islands represent a distinct epidemiological setting compared with mainland Spain because of their subtropical climate and their strong transport connections with Africa and Latin America, factors that may facilitate the introduction of invasive mosquito species. The detection of this species is of particular concern because of its high vector competence and its strong adaptation to human environments [17]. *Ae. japonicus*, another invasive species originally from East Asia, has also been reported in several European countries and has recently been detected in northern regions of Spain [18].

The establishment of competent vectors in Europe increases the potential risk of local transmission of arboviruses, particularly in areas with high levels of international travel from endemic regions. Imported cases of dengue, chikungunya, and Zika are reported regularly in European countries, and when these cases occur in areas where competent mosquito vectors are present, the possibility of autochthonous transmission arises [19]. Although large outbreaks remain uncommon in Europe, several episodes of local transmission have been reported in recent years, highlighting the importance of strengthening surveillance systems and vector control strategies [7,20,21,22,23,24,25,26,27,28,29].

Climate change has emerged as one of the most important drivers of invasive mosquito expansion in Europe. Rising temperatures, changes in precipitation patterns, prolonged transmission seasons, and increasing urbanization enhance habitat suitability for invasive *Aedes* species and may facilitate both vector establishment and arboviral transmission. Evidence from Mediterranean Europe suggests that these environmental changes are likely to increase the future burden of mosquito-borne diseases in the region [14].

To address these risks, European countries have developed surveillance systems aimed at monitoring the presence and distribution of invasive mosquito species. In Spain, entomological surveillance is coordinated through national and regional public health authorities and includes monitoring programs designed to detect the introduction and expansion of invasive vectors. The Health Alerts and Emergencies Coordination Centre (CCAES) of the Spanish Ministry of Health plays a key role in assessing public health risks related to vector-borne diseases and coordinating response strategies.

Understanding the distribution and expansion dynamics of invasive mosquito species is essential for improving risk assessment and designing effective prevention strategies. Early detection of new vector populations allows public health authorities to implement control measures and reduce the probability of arbovirus transmission.

The aim of this review is to analyse the historical expansion of invasive *Aedes* mosquitoes in Spain from their first introduction through March 2026, critically evaluate existing surveillance systems, identify factors associated with successful establishment, and assess implications for preparedness against arbovirus emergence. In particular, this work focuses on the geographical distribution of *Ae. albopictus*, *Ae. aegypti*, and *Ae. japonicus*, as well as the environmental and socioeconomic factors that facilitate their establishment. The findings contribute to a better understanding of vector surveillance in Spain and provide relevant information for strengthening preparedness against vector-borne diseases.

To our knowledge, this is the first review specifically focused on Spain that integrates official surveillance reports, European Centre for Disease Prevention and Control (ECDC) and VectorNet information, citizen science evidence from Mosquito Alert, invasion genetics findings, and comparative surveillance preparedness assessment, providing a comprehensive evaluation of invasive *Aedes* surveillance and arbovirus preparedness.

## 2. Methods

### 2.1. Study Design

This study was conducted as a narrative review with a descriptive surveillance analysis focusing on the detection and distribution of invasive mosquito species of the genus *Aedes* in Spain. The methodological framework followed principles commonly applied in scoping reviews in public health research, allowing the identification and synthesis of heterogeneous sources including surveillance reports, institutional documents, and peer-reviewed scientific literature [30,31]. The reporting structure of the review was informed by the PRISMA Extension for Scoping Reviews (PRISMA-ScR) guidelines to ensure transparency in the identification, selection, and synthesis of relevant sources [32].

### 2.2. Information Sources

The main sources of information included official surveillance reports issued by national and international public health institutions, as well as peer-reviewed scientific publications. Institutional sources included documents from the ECDC, the VectorNet project, the World Health Organization (WHO), and reports published by the CCAES.

Scientific literature was retrieved from major bibliographic databases, including PubMed/MEDLINE, Scopus, and Web of Science. Additional information was obtained from grey literature such as epidemiological bulletins, surveillance reports, and documents published by national and regional public health authorities. These sources are frequently used in studies addressing emerging vector-borne diseases and invasive mosquito surveillance [33,34].

### 2.3. Search Strategy

The literature search aimed to identify documents published between January 2004 and March 2026 that addressed the detection, distribution, surveillance, or epidemiological relevance of invasive *Aedes* mosquito species in Spain or Europe. The search period started in January 2004 because this corresponds to the year of the first documented detection of *Ae. albopictus* in Spain, which marked the beginning of invasive *Aedes* establishment in the country and provides a relevant baseline for evaluating subsequent expansion and surveillance activities. The search included combinations of keywords related to mosquito vectors and arbovirus transmission.

Example search strings included:

*Aedes* AND (albopictus OR aegypti OR japonicus)

AND (Spain OR Europe)

AND (surveillance OR distribution OR invasive species)

Scopus/Web of Science:

TITLE-ABS-KEY (*Aedes* AND invasive AND (Spain OR Europe))

AND (vector surveillance OR distribution OR mosquito)

Additional searches were conducted using Google Scholar and institutional repositories to identify relevant surveillance reports and technical documents.

### 2.4. Study Selection Process

**Figure 1:**
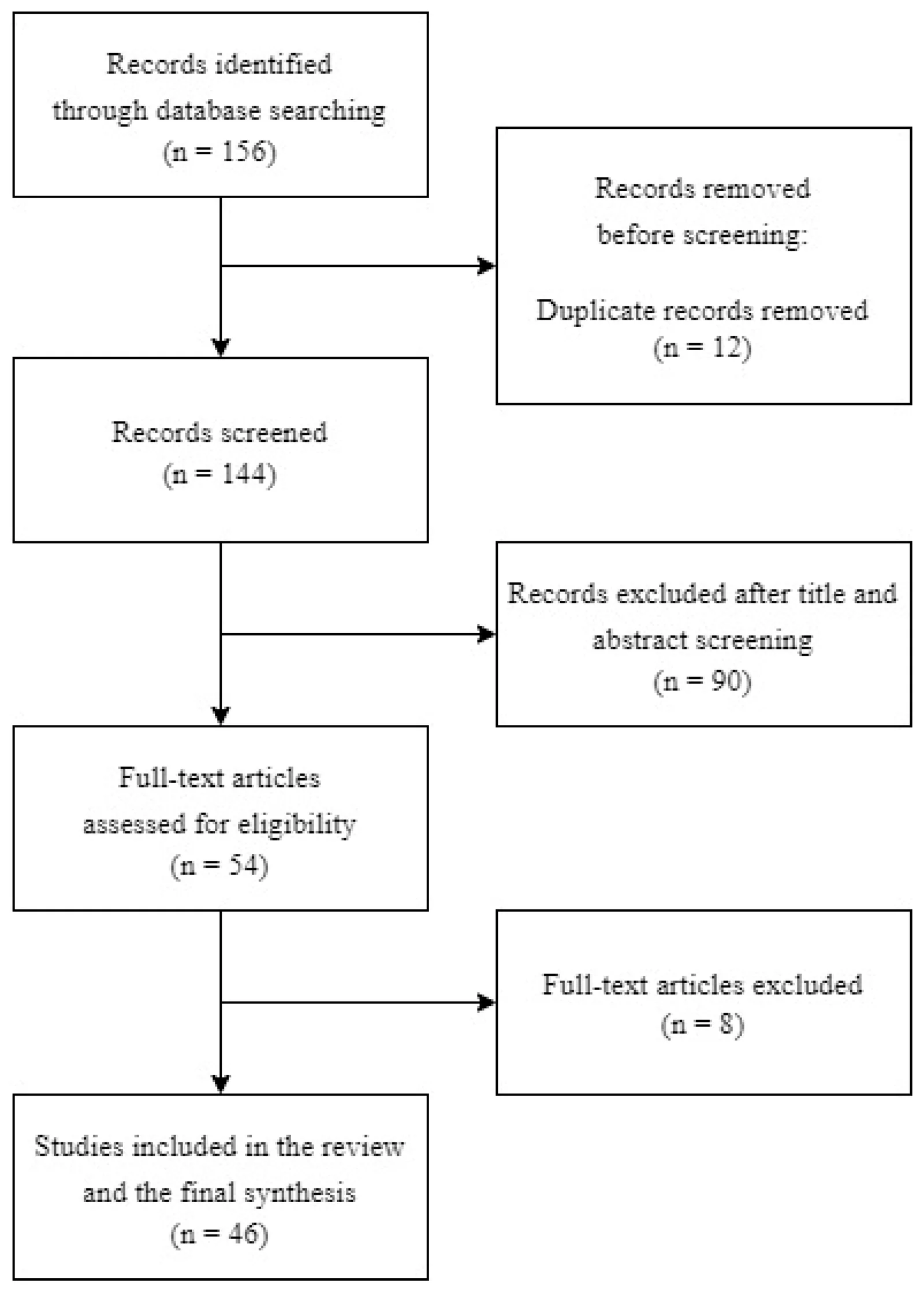
PRISMA flow diagram illustrating the identification, screening, eligibility assessment and inclusion of studies in the review.

All references retrieved through database searches were exported to a reference management software and screened in two stages. First, titles and abstracts were reviewed to assess their relevance according to the eligibility criteria. Subsequently, full texts of potentially relevant documents were evaluated. Duplicates were removed prior to screening. Reasons for exclusion at the full-text stage were documented and are presented in the PRISMA flow diagram (Figure 1).

The search identified 156 records. After removal of duplicates, 144 records underwent title and abstract screening. A total of 54 full-text documents were assessed, of which 46 met the eligibility criteria and were included in the final synthesis.

Full-text articles excluded (n = 8): Did not address invasive *Aedes* surveillance (n = 3); Focused exclusively on imported cases (n = 2); Insufficient geographical relevance (n = 2); Duplicate information/source superseded by updated report (n = 1).

### 2.5. Eligibility Criteria

Documents were considered eligible if they met at least one of the following criteria:

- Reported on the detection or geographical distribution of invasive *Aedes* species in Spain or Europe;
- Described entomological surveillance systems or monitoring programmes;
- Analysed environmental or epidemiological factors associated with the expansion of invasive mosquito vectors; or
- Addressed public health responses to the introduction of invasive *Aedes* species.

The review included both peer-reviewed scientific publications and official institutional reports. Documents exclusively addressing imported arboviral cases without reference to vector presence or surveillance were excluded.

### 2.6. Data Extraction and Synthesis

Information extracted from each source included the mosquito species reported, geographical location of detection, year of detection, surveillance methods used, and relevant public health implications. Data were analysed descriptively to identify patterns of vector expansion, geographical distribution, and surveillance activities.

Included sources were also classified according to their type of evidence. Sources were grouped into five categories: peer-reviewed scientific publications, official surveillance reports, institutional technical documents, epidemiological bulletins, and other grey literature. This classification was used to characterize the evidence base supporting the review and to evaluate the relative contribution of scientific and institutional sources.

Of the 46 sources included in the final synthesis, 37 (80%) were peer-reviewed publications and 9 (20%) corresponded to grey literature, including surveillance reports, technical documents and epidemiological bulletins.

Drivers of invasive *Aedes* expansion were identified through an inductive thematic analysis of the reviewed literature. Factors repeatedly associated with mosquito introduction, establishment, or geographical spread were extracted and classified into four major categories: climate change, tourism and human mobility, international trade, and urbanisation. The frequency with which each factor was reported across the reviewed sources was recorded and used to construct Table 2.

The synthesis focused particularly on the distribution of *Ae. albopictus*, *Ae. aegypti*, and *Ae. japonicus* in Spain, as well as on environmental, climatic, and socioeconomic factors that may facilitate their establishment. These findings were integrated to provide an overview of current surveillance strategies and their relevance for preventing the transmission of arboviruses in Spain.

### 2.7. Quality Assessment

A formal risk-of-bias assessment was not performed because the review included heterogeneous evidence sources, such as surveillance reports, institutional documents, and peer-reviewed literature. This approach is consistent with scoping review methodology aimed at mapping available evidence rather than quantitatively assessing study quality.

## 3. Results

### 3.1. Distribution of Invasive Aedes Species in Spain

Surveillance data and published literature indicate that invasive mosquito species of the genus *Aedes* have progressively expanded their distribution across Spain during the past two decades. Among these species, *Ae. albopictus* is currently the most widespread and epidemiologically relevant vector.

The first confirmed detection of *Ae. albopictus* in Spain occurred in Catalonia in 2004, marking the beginning of the species’ establishment in the Iberian Peninsula [15]. Since then, the species has expanded progressively along the Mediterranean coastline, where climatic conditions and urban environments provide favourable habitats for mosquito breeding. As summarised in Table 1, *Ae. albopictus* is currently established in several autonomous communities, including Catalonia, the Valencian Community, Murcia, the Balearic Islands and parts of Andalusia [16,34].

**Figure 2:**
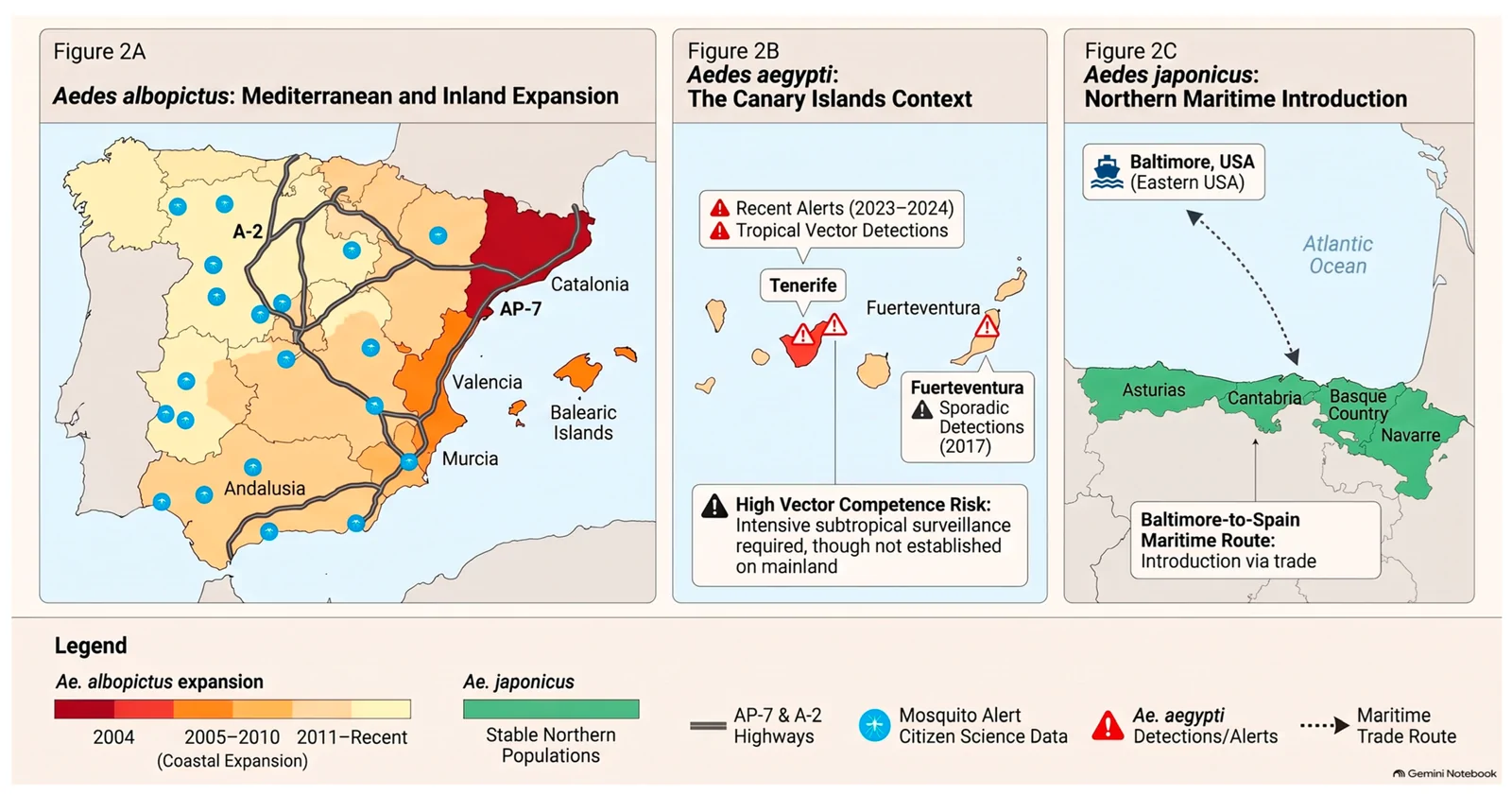
Geographic Distribution and Invasion Dynamics of Invasive *Aedes* Mosquitoes in Spain.

The geographical distribution of invasive *Aedes* mosquito detections in Spain is illustrated in Figure 2, which highlights the concentration of established *Ae. albopictus* populations along Mediterranean coastal areas and the occurrence of sporadic detections in other regions. Recent surveillance reports also suggest that the distribution of *Ae. albopictus* has gradually expanded into inland territories, indicating increasing ecological adaptability of the species.

### 3.2. Detection of Other Invasive Aedes Species

In addition to *Aedes albopictus*, other invasive mosquito species have been detected sporadically in Spain. As shown in Table 1, *Ae. aegypti*, the primary vector of dengue worldwide, has been detected on several occasions in the Canary Islands. Although these detections have raised public health concerns, there is currently no evidence of stable populations established in mainland Spain [34].

Another invasive species, *Ae. japonicus*, has recently been detected in northern regions of Spain. This species, originally native to East Asia, has expanded across several European countries during the past two decades. Surveillance studies have confirmed its presence in northern Spain, suggesting that environmental conditions may support the establishment of this species in temperate regions [18]. While the epidemiological importance of *Ae. japonicus* remains lower than that of *Ae. albopictus*, its detection underscores the importance of maintaining continuous surveillance programmes capable of identifying emerging vector species.

New genetic evidence has provided important insights into the invasion dynamics of *Aedes japonicus* in Spain. A large-scale population genetics study conducted across Europe, the United States and Japan found that Spanish populations form a distinct genetic cluster and are predominantly distributed along the northern coast, including Asturias, Cantabria, the Basque Country and Navarre. The study suggests that the most likely introduction route into Spain was maritime transport from the eastern United States, particularly from the Baltimore area, followed by local establishment and expansion in northern Spanish regions. These findings highlight the importance of ports of entry as critical points for invasive mosquito surveillance and early detection activities [35].

European surveillance data indicate that the invasion process is not limited to *Ae. albopictus* and *Ae. japonicus*. The continued expansion of *Ae. koreicus* and the re-emergence of *Ae. aegypti* in several Mediterranean territories reinforce the need for continuous surveillance programmes capable of detecting new introductions before widespread establishment occurs. These observations suggest that vector surveillance systems should address multiple invasive mosquito species simultaneously rather than focusing on a single species [14].

### 3.3. Environmental and Socioeconomic Drivers of Vector Expansion

Several environmental and socioeconomic factors contribute to the introduction and spread of invasive mosquito species in Spain. The main drivers of vector expansion identified in this study are summarised in Table 2. Climatic conditions, particularly temperature and precipitation patterns, play a critical role in determining the suitability of habitats for mosquito survival and reproduction [6,8].

The evidence identified corresponds to the number of reviewed sources reporting an association between each driver and the introduction, establishment, or geographic expansion of invasive *Aedes* species.

Relevance to Spain was qualitatively classified according to the frequency and consistency with which each factor was identified in the reviewed literature. High relevance indicates factors repeatedly reported as major drivers of invasive mosquito establishment and spread in Spain. Moderate–High relevance reflects factors supported by substantial evidence but with a less direct or less consistent influence across studies, whereas Moderate relevance indicates factors reported less frequently or with more limited supporting evidence.

Climate change has been identified as an important factor facilitating the northward expansion of mosquito vectors across Europe. Increasing temperatures may extend the seasonal activity period of mosquito populations and enable the establishment of species in regions that were previously unsuitable [4,6,8].

Temperature is one of the most important environmental determinants influencing mosquito population dynamics and arbovirus transmission. Higher temperatures may accelerate mosquito development, increase feeding frequency, shorten pathogen incubation periods, and extend seasonal vector activity. In addition, warmer winters may facilitate overwintering survival and enable the establishment of invasive mosquito populations in areas previously considered climatically unsuitable [14].

Human mobility and international trade also represent major pathways for the introduction and spread of invasive mosquito species [3,7,8]. Furthermore, passive dispersal of adult *Ae. albopictus* by motor vehicles has been documented in Spain, providing direct evidence that road transport contributes to the expansion of established populations and facilitates the colonisation of new areas [5].

Spain’s high levels of international tourism and trade further increase the probability of both vector introduction and the arrival of infected travellers carrying arboviruses. These conditions create favourable circumstances for the occurrence of local transmission events in areas where competent mosquito vectors are already present.

In addition to climatic and anthropogenic drivers, the biological characteristics of *Ae. albopictus* may contribute substantially to its invasion success. A comprehensive review conducted in the Americas highlighted the species’ ability to feed on multiple mammalian and avian hosts and documented natural infections with several arboviruses, including dengue, Zika, West Nile, La Crosse, Eastern equine encephalitis, and yellow fever viruses. Such ecological flexibility may enhance its capacity to establish and persist in newly invaded territories [9].

### 3.4. Surveillance Systems and Monitoring Activities

Spain has developed multiple surveillance initiatives aimed at monitoring invasive mosquito populations and assessing the associated risk of vector-borne diseases, coordinated by the CCAES. The evolution of mosquito detections in Spain is summarised in Table 1, which outlines key milestones in the detection of invasive *Aedes* species.

These surveillance programmes involve collaboration between national, regional and local public health authorities. Entomological monitoring networks collect data on mosquito distribution, abundance and seasonal activity patterns, enabling the early detection of invasive mosquito populations.

At the European level, initiatives such as the VectorNet project provide updated maps of vector distribution and support cross-border surveillance activities [36]. In Spain, the CCAES plays a central role in evaluating the public health implications of invasive mosquito detections and coordinating response strategies when necessary.

Together, these surveillance systems contribute to improving early detection capabilities and provide essential information for guiding public health interventions aimed at reducing the risk of arbovirus transmission.

The detection and subsequent expansion of *Ae. japonicus* in northern Spain further illustrates the importance of maintaining active surveillance programmes capable of identifying new invasion events and monitoring mosquito spread in real time [35].

Contemporary surveillance approaches increasingly adopt a One Health perspective integrating entomological, human, animal, and environmental data. This approach has proven particularly valuable in European mosquito-borne disease surveillance programmes because it allows earlier detection of emerging threats and supports better-informed public health responses. The integration of surveillance data from multiple sectors may improve preparedness against future arboviral outbreaks [14].

### 3.5. Citizen Science and Mosquito Alert as a Complementary Surveillance Tool

In addition to conventional entomological surveillance programmes coordinated by national and regional public health authorities, citizen science initiatives have emerged as valuable complementary tools for the early detection of invasive mosquito species. Among these, the Mosquito Alert platform represents one of the most prominent examples in Europe. Developed in Spain, Mosquito Alert enables citizens to report mosquito sightings through a smartphone application, generating georeferenced data that are subsequently validated by entomology experts [37,38].

Over the last decade, Mosquito Alert has contributed substantially to the surveillance of *Aedes albopictus* and other invasive mosquito species by increasing the spatial coverage of monitoring activities beyond areas routinely surveyed by public health services. The platform has facilitated the identification of new mosquito occurrences, improved public awareness regarding vector-borne diseases, and supported collaboration between researchers, public health professionals, and citizens [37,38].

Citizen-generated records from Mosquito Alert have contributed to the detection and geographical monitoring of *Ae. albopictus* across Spain and have helped identify mosquito occurrences beyond areas routinely covered by institutional surveillance programmes. The broad geographic coverage achieved through public participation complements official surveillance systems and may facilitate the identification of new invasion fronts and priority areas for entomological investigation. By expanding surveillance reach and supporting early detection activities, citizen science initiatives can strengthen preparedness and improve the timeliness of public health responses to invasive mosquito introductions.

Citizen-generated data may be particularly useful for the early detection of invasive species in areas where institutional surveillance is limited. In Spain, where surveillance intensity varies among autonomous communities, citizen science initiatives can provide additional information that helps identify potential introductions and guides targeted field investigations. Furthermore, the integration of citizen science data with traditional entomological monitoring may enhance the capacity to detect changes in mosquito distribution patterns and support rapid public health responses [39].

Nevertheless, citizen science approaches also present important limitations. Reporting rates are influenced by public participation, digital literacy, and population density, potentially generating geographical biases in data collection. In addition, observations require expert validation to minimise misclassification of mosquito species. Therefore, citizen science should be considered a complementary component of integrated vector surveillance rather than a substitute for systematic entomological monitoring [39,40].

Overall, the experience of Mosquito Alert demonstrates the potential value of public participation in strengthening invasive mosquito surveillance in Spain, and highlights the importance of integrating innovative digital tools into future preparedness strategies for arbovirus prevention.

These observations demonstrate that citizen science can contribute not only to public awareness but also to the early detection and geographical monitoring of invasive mosquito species, thereby complementing conventional surveillance systems.

## 4. Discussion

The results of this study confirm the progressive expansion of *Ae. albopictus* across Spain, particularly along Mediterranean coastal regions and increasingly into inland territories. The observed distribution pattern is consistent with evidence identifying climatic suitability, urbanisation, tourism, international mobility, and trade as major drivers of invasive *Aedes* establishment and spread. Similar expansion trends have been reported in other Mediterranean European countries where *Ae. albopictus* has successfully colonised large geographical areas following its introduction [16,33].

Nevertheless, interpretation of distribution patterns should consider regional differences in surveillance intensity, which may influence the probability of species detection and reporting.

The Spanish experience mirrors that of other Mediterranean countries where the establishment of *Ae. albopictus* has been followed by an increasing risk of autochthonous arbovirus transmission. In Italy, for example, the expansion of this species has been associated with outbreaks of chikungunya and dengue, highlighting its epidemiological relevance for public health preparedness in Europe [41].

More recent evidence further demonstrates the increasing public health relevance of *Ae. albopictus* in Europe. Autochthonous outbreaks of dengue and chikungunya continue to occur in Mediterranean countries, confirming that Europe can no longer be regarded solely as a region affected by imported arboviral infections. The interaction between expanding vector populations, imported viraemic travellers, and increasingly favourable climatic conditions creates a scenario in which local transmission events may become more frequent in the coming decades [14].

Similarly, several episodes of autochthonous arbovirus transmission have been reported in southern France in recent years, illustrating how the presence of competent vectors combined with imported cases can lead to local transmission events [7,20,21,22,23,24,26,27,28,29].

Although *Ae. albopictus* remains the most widespread invasive mosquito species in Spain, the detection of other *Aedes* species further emphasises the importance of maintaining comprehensive surveillance systems. As summarised in Table 1, sporadic detections of *Ae. aegypti* have occurred in the Canary Islands. The epidemiological significance of these detections should be interpreted in the context of the Canary Islands, which differ from mainland Spain in terms of climatic suitability and international connectivity. This species is considered the primary global vector of dengue and several other arboviruses, and its presence in Spain, even if temporary, represents a potential public health concern due to its high vector competence and strong adaptation to urban environments [8,17,42].

The recent detection of *Ae. japonicus* in northern Spain also illustrates the ongoing dynamics of mosquito invasions in Europe. This species has expanded across several European countries over the last two decades and has demonstrated a capacity to establish populations in temperate climates [18]. Although its role in arbovirus transmission is considered less significant than that of *Ae. albopictus*, its presence highlights the need for continuous monitoring of emerging vector species.

Population genetic analyses have substantially improved our understanding of the emergence of *Ae. japonicus* in Spain. Unlike the gradual expansion pattern described for *Ae. albopictus*, evidence suggests that Spanish *Ae. japonicus* populations may have originated from a long-distance introduction event linked to maritime trade routes. The apparent genetic relationship between Spanish populations and populations from the eastern United States reinforces the role of international trade and ports of entry as key drivers of invasive mosquito introductions, highlighting the need to strengthen surveillance activities in these strategic locations [35].

The epidemiological importance of *Ae. albopictus* extends beyond its expanding geographical distribution. A recent review of evidence from the Americas demonstrated that this species could participate in both horizontal and vertical transmission of dengue virus and may contribute to virus maintenance during inter-epidemic periods. The authors reported similar minimum infection rates for dengue virus between horizontal and vertical transmission pathways, suggesting that *Ae. albopictus* may play a more relevant role in arbovirus ecology than traditionally assumed. These findings reinforce the need for surveillance systems to monitor not only mosquito distribution but also viral circulation within vector populations [9].

The expansion of invasive mosquito species in Spain is driven by multiple environmental and socioeconomic factors, summarised in Table 2. Climatic conditions play a key role in determining the suitability of habitats for mosquito survival and reproduction. Climate change has been widely recognised as a major driver influencing the geographical distribution of mosquito vectors worldwide. Increasing temperatures may extend the seasonal activity of mosquito populations and facilitate the establishment of invasive species in regions that were previously unsuitable [4,6,43].

Several modelling studies have suggested that climate change may lead to the northward expansion of *Aedes* mosquitoes across Europe in the coming decades, potentially increasing the geographical range of arbovirus transmission risk [6,44,45]. These projections highlight the importance of strengthening surveillance and preparedness strategies in European countries where invasive mosquito species are already present.

In addition to climatic drivers, globalisation and international trade have played a crucial role in the spread of invasive mosquito species. Spain’s position as a major tourist destination and international trade hub increases the likelihood of both vector introduction and the arrival of travellers infected with arboviruses [3,7,8].

The chronological evolution of mosquito detections in Spain, summarised in Table 1, also highlights the importance of surveillance systems for identifying emerging vector populations. Entomological monitoring networks provide essential information for assessing vector distribution, abundance and seasonal dynamics. Early detection of invasive mosquito populations allows public health authorities to implement targeted control measures and reduce the probability of arbovirus transmission.

At the European level, initiatives such as the VectorNet programme coordinated by the ECDC play a key role in improving the surveillance of invasive mosquito species and facilitating cross-border collaboration [36]. These initiatives provide updated vector distribution maps and support risk assessments for vector-borne diseases across Europe.

### 4.1. Comparison of Surveillance Approaches in Spain and Other European Countries

Spain shares many of the surveillance challenges observed in other European countries affected by the expansion of invasive *Aedes* mosquitoes. Italy, France and Spain represent three of the European countries with the widest establishment of *Aedes albopictus* populations and the greatest documented risk of autochthonous arbovirus transmission. However, important differences exist in the organisation and implementation of surveillance activities. In Italy, surveillance systems have expanded in response to several outbreaks of chikungunya and dengue, leading to the development of regional monitoring programmes and outbreak response protocols. Similarly, France has strengthened vector surveillance through coordinated national frameworks and extensive monitoring in areas where autochthonous transmission has previously occurred. The increasing frequency of locally acquired dengue and chikungunya cases in Mediterranean Europe has reinforced the importance of integrated vector surveillance and preparedness strategies across affected countries [14].

In Spain, surveillance activities are coordinated through the CCAES and regional public health authorities. Although substantial progress has been achieved over the last decade, surveillance intensity remains heterogeneous among autonomous communities, with some regions maintaining more extensive entomological monitoring networks than others. This variability may influence detection capacity and complicate direct comparisons of vector distribution across the country. Strengthening harmonised surveillance protocols and improving data sharing between regional and national authorities could enhance the consistency of surveillance outputs and facilitate early detection of emerging vector populations [14].

Another distinctive feature of the Spanish context is the integration of citizen science initiatives such as Mosquito Alert, which has become one of the most developed community-based surveillance programmes in Europe. The combination of traditional entomological surveillance, citizen-generated data, and national risk assessment frameworks provides opportunities for improving early warning capacity. Future surveillance strategies may benefit from greater incorporation of digital epidemiology tools, predictive modelling approaches and One Health surveillance systems that integrate environmental, animal and human health data. Similar recommendations have recently been proposed for Mediterranean European countries facing increasing arbovirus risks under climate change scenarios [14].

The contribution of Mosquito Alert extends beyond public engagement and provides a practical surveillance advantage by increasing geographical coverage and supporting the early detection of invasive mosquito occurrences. Citizen-generated observations, once validated by entomology experts, can complement conventional surveillance programmes by identifying mosquito presence in areas where routine monitoring is limited or absent. This additional source of information may facilitate the recognition of emerging invasion fronts and strengthen early warning capacity within the Spanish surveillance framework.

To place the Spanish surveillance framework into a broader European context, a comparison with France and Italy is particularly informative. These countries share similar climatic conditions, established populations of *Aedes albopictus*, and documented episodes of autochthonous arbovirus transmission, making them useful benchmarks for evaluating surveillance capacity and preparedness. Although all three countries have strengthened vector surveillance systems during the last two decades, differences remain in the level of national coordination, integration of citizen science approaches, outbreak response experience, and implementation of entomological monitoring programmes. As summarised in Table 3, comparing these surveillance frameworks helps identify strengths and gaps within the Spanish system and highlight opportunities for improving preparedness against future arboviral threats in Mediterranean Europe [14,22,26,27,36,37,38,39,40].

The experience of Mosquito Alert illustrates the potential of citizen science to complement conventional entomological surveillance. Although citizen-generated observations require expert validation and may be affected by reporting bias, they can substantially increase surveillance coverage and facilitate early detection of new mosquito introductions. The formal integration of validated citizen science data into national surveillance frameworks could strengthen early warning systems and improve preparedness against arbovirus emergence in Spain.

While surveillance remains a fundamental component of preparedness, vector control programmes increasingly face challenges associated with insecticide resistance. Resistance-associated mutations have already been reported in European populations of *Aedes albopictus* and *Culex pipiens*, potentially reducing the effectiveness of conventional control measures based on pyrethroid insecticides. Consequently, integrated vector management strategies incorporating environmental management, biological control methods, and innovative technologies should be prioritized [14].

Despite the increased theoretical risk of arbovirus transmission associated with the presence of invasive mosquito species, sustained local transmission remains relatively uncommon in Europe. The occurrence of outbreaks depends on multiple interacting factors, including vector density, environmental conditions, and the timely identification of imported cases [19,46]. In Spain, most reported cases of arboviral infections are currently imported cases associated with international travel, although sporadic local transmission events have been documented [20,21,24].

For this reason, strengthening vector surveillance and improving coordination between public health institutions remain essential priorities. Integrated vector management strategies combining environmental management, entomological monitoring and public awareness campaigns may contribute to reducing mosquito breeding sites and limiting the expansion of invasive populations.

Overall, the continued expansion of *Aedes* mosquito species in Spain underscores the need for sustained surveillance efforts and adaptive public health strategies. Improved monitoring systems, enhanced coordination between national and European institutions, and increased research on vector ecology will be essential for mitigating the potential impact of vector-borne diseases in Spain and across Europe. The key recommendations for strengthening invasive *Aedes* mosquito surveillance and public health preparedness in Spain are summarised in Table 4.

Another growing challenge for vector control programmes is the emergence of insecticide resistance. Several European populations of *Aedes* albopictus and Culex pipiens have already shown resistance-associated mutations to pyrethroids and other compounds commonly used in public health interventions. This situation may compromise the long-term effectiveness of conventional vector control strategies and highlights the need to strengthen integrated vector management programmes based on sustainable and evidence-based approaches [14].

### 4.2. Public Health Response and Vector Control Strategies

The expansion of invasive *Aedes* mosquitoes requires not only effective surveillance systems but also well-established response mechanisms capable of reducing the risk of arbovirus transmission once vector populations are detected. In Spain, public health preparedness relies on a combination of entomological surveillance, rapid epidemiological investigation of imported and autochthonous cases, and vector control interventions coordinated by regional authorities within the framework of national risk assessment protocols. These measures aim to prevent the establishment of transmission cycles following the introduction of viraemic travellers into areas where competent vectors are already present [14,19,36].

Current vector control strategies are primarily based on Integrated Vector Management (IVM) approaches. These include source reduction through the elimination of artificial breeding sites, larval control interventions in areas with persistent mosquito activity, and targeted adult mosquito control measures when epidemiological risk assessments indicate a significant transmission threat. Environmental management remains particularly important for *Aedes albopictus*, whose breeding habitats are largely associated with urban and peri-urban containers, such as flowerpots, water storage containers, and discarded tyres [3,14,33,41].

When imported or autochthonous arboviral cases are identified, rapid response activities should include intensified entomological surveillance within the affected area, assessment of local vector abundance, and implementation of targeted control measures around probable exposure locations. Such interventions are especially relevant in Mediterranean regions where established populations of *Aedes albopictus* coincide with high levels of international travel and tourism. Experience from Spain, France and Italy demonstrates that early detection combined with rapid vector control can contribute to limiting the likelihood of onward transmission following viral introduction [20,21,22,23,24,25,26,27,28,29,36].

Emerging technologies may further strengthen preparedness capacities. The integration of digital epidemiology tools, real-time reporting platforms, predictive ecological suitability models and artificial intelligence-based risk forecasting systems could improve the identification of areas at increased risk of vector establishment and arbovirus transmission [14,37,38,39,40].

Future preparedness strategies should also consider the growing challenge of insecticide resistance and the evaluation of innovative control methods. Approaches such as *Wolbachia*-based interventions, the Sterile Insect Technique (SIT), and other environmentally sustainable control technologies are increasingly being explored as complementary alternatives to conventional insecticide-based programmes. The incorporation of these strategies within a coordinated One Health framework may improve long-term resilience against emerging arboviral threats in Spain and across Mediterranean Europe [14,47,48,49].

### 4.3. Outbreak Response and Vector Control Measures

In addition to routine surveillance activities, Spain has established response protocols aimed at preventing onward transmission when imported or autochthonous arboviral cases are detected. The CCAES, in collaboration with regional public health authorities, coordinates risk assessment and recommends control measures according to the epidemiological situation and the presence of competent vectors. These actions are integrated within the national preparedness framework for vector-borne diseases and seek to interrupt local transmission cycles before wider spread occurs.

When imported or autochthonous arboviral cases are detected, response activities include intensified entomological investigations and targeted vector control interventions aimed at reducing transmission risk. These measures are implemented within an Integrated Vector Management framework and are adapted according to local epidemiological circumstances and vector abundance [14,47,48].

Rapid implementation of these measures is particularly important in Mediterranean regions of Spain, where established populations of *Aedes albopictus* coexist with intense tourism and international travel. Experiences from Spain and other European countries affected by autochthonous dengue and chikungunya transmission have demonstrated that early case detection, prompt epidemiological investigation, and rapid vector control interventions represent critical components of outbreak containment strategies [20,21,22,23,24,25,26,27,28,29].

Finally, the growing challenge of insecticide resistance highlights the need to complement conventional outbreak response measures with innovative and sustainable approaches. Future preparedness plans should strengthen integrated vector management strategies incorporating environmental control, citizen participation, digital surveillance tools, and emerging technologies such as *Wolbachia*-based interventions and the Sterile Insect Technique (SIT), consistent with One Health approaches that are increasingly recommended for arbovirus prevention and control in Europe [14,49].

## 5. Limitations

This study has several limitations. First, the review relied on surveillance reports, grey literature, and published scientific studies that may differ in methodological quality, geographical coverage, and reporting standards. Second, surveillance intensity is not homogeneous across Spain. Some autonomous communities, particularly those along the Mediterranean coast, have implemented more extensive entomological monitoring programmes than other regions. Consequently, the apparent concentration of mosquito detections in these areas may partly reflect greater detection effort rather than true differences in mosquito distribution or abundance. Third, the inclusion of grey literature and institutional reports may introduce variability in the level of detail available for data extraction. Finally, as a narrative review with a descriptive surveillance component, the study does not allow causal inference regarding the relative contributions of climate, mobility, trade, or urbanisation to mosquito expansion.

Publication bias may also have influenced the available evidence, as regions with more established surveillance programmes are more likely to generate scientific publications and official reports. Consequently, some areas with lower surveillance activity may be underrepresented.

Furthermore, the absence of a formal risk-of-bias assessment limits direct comparison of the methodological quality of the included sources. Findings should therefore be interpreted as a synthesis of the available evidence rather than as a quantitative evaluation of study quality.

## 6. Conclusions

The continued expansion of *Aedes albopictus* and the emergence of other invasive mosquito species such as *Aedes japonicus* highlight the need to maintain invasive mosquito surveillance as a public health priority in Spain. Future surveillance systems should adopt integrated One Health approaches that combine conventional entomological monitoring, citizen science initiatives, environmental surveillance, and digital epidemiology tools.

Compared with other Mediterranean European countries such as France and Italy, Spain has made substantial advances in invasive mosquito surveillance through the combination of institutional monitoring systems, national risk assessments and citizen science initiatives. Nevertheless, further harmonisation between autonomous communities, broader integration of innovative surveillance approaches and stronger inter-regional coordination remain important priorities for future preparedness against arboviral threats.

The increasing influence of climate change, urbanization, globalization, and international mobility is likely to enhance the suitability of European territories for invasive mosquito establishment and arbovirus transmission. In this context, strengthening harmonized surveillance activities across autonomous communities, improving inter-institutional coordination, and enhancing early warning systems will be essential for preparedness against future arboviral outbreaks.

Furthermore, the development and evaluation of innovative vector control tools, including Wolbachia-based strategies and the Sterile Insect Technique (SIT), may provide sustainable alternatives to conventional insecticide-based control programmes and contribute to long-term public health preparedness.

## Figures and Tables

**Table 1:** Chronology, expansion milestones and current distribution of invasive *Aedes* species in Spain.

Species	First Detection in Spain	Main Expansion Milestones	Current Distribution/Status
*Ae. albopictus*	2004, Catalonia (San Cugat del Vallés)	Valencian Community (2005–2010), Murcia (2011), Balearic Islands, Andalusia, progressive inland spread	Established in multiple autonomous communities
*Ae. aegypti*	Fuerteventura (2017)	Fuerteventura (2017), Tenerife (2023–2024 alerts), additional detections under surveillance	Not established in mainland Spain
*Ae. japonicus*	Asturias/Cantabria	Asturias, Cantabria, Basque Country and Navarre	Emerging and locally established

**Table 2:** Evidence-based environmental and socioeconomic drivers of invasive *Aedes* mosquito expansion in Spain identified through the literature review.

Driver	Evidence Identified (n Studies)	Mechanism	Relevance to Spain	Key References
Climate change	11	Increased thermal suitability	High	[4,5,35,36,37]
Tourism	8	Imported cases	High	[16,17,18,21]
Trade	9	Egg introduction	Moderate–High	[3,6,7,33]
Urbanisation	6	Breeding habitats	High	[7,13,34]

**Table 3:** Comparative overview of invasive *Aedes* mosquito surveillance systems in Spain, France and Italy. The comparison is based on evidence from European surveillance reports, VectorNet information, published outbreak investigations and recent reviews addressing vector-borne disease preparedness in Mediterranean Europe [14,22,26,27,36,37,38,39,40].

Feature	Spain	France	Italy
National coordination	Coordinated by the Health Alerts and Emergencies Coordination Centre (CCAES) together with regional public health authorities. Surveillance responsibilities are shared across autonomous communities.	Coordinated through national public health and vector surveillance frameworks with strong involvement of regional authorities in areas affected by autochthonous transmission.	National and regional authorities collaborate through established vector surveillance and outbreak response programmes developed after chikungunya and dengue outbreaks.
Entomological surveillance	Extensive surveillance programmes implemented in several autonomous communities, although monitoring intensity remains heterogeneous across regions.	Long-standing surveillance systems in southern regions where *Aedes albopictus* is established, including routine vector monitoring and rapid response activities.	Well-established monitoring networks covering regions affected by invasive mosquito expansion and previous arboviral outbreaks.
Citizen science initiatives	Strong integration of Mosquito Alert, one of the largest citizen science platforms for mosquito surveillance in Europe.	Citizen participation initiatives exist but are less integrated at national level than Mosquito Alert.	Citizen science approaches are used in some regions but remain less prominent than institutional surveillance programmes.
Autochthonous arbovirus transmission	Autochthonous dengue outbreaks reported, including the first documented outbreak in mainland Spain and subsequent transmission events.	Multiple autochthonous dengue and chikungunya transmission events reported in recent years.	Several chikungunya and dengue outbreaks have occurred since the establishment of *Aedes albopictus*, contributing to strengthened surveillance and preparedness measures.
Main challenges	Regional heterogeneity in surveillance intensity; harmonisation of surveillance protocols; integration of digital tools and predictive systems.	Sustaining surveillance over extensive territories with established vector populations; adapting surveillance to climate-driven expansion.	Controlling established mosquito populations and responding rapidly to recurrent arboviral outbreaks.
Future priorities	Greater inter-regional coordination, integration of citizen science, digital epidemiology and One Health surveillance approaches.	Strengthening preparedness for increasing arbovirus transmission risk under climate change scenarios.	Enhancing integrated vector management and maintaining long-term surveillance capacity.
Main strength	Mosquito Alert integration	National coordination	Outbreak experience
Main limitation	Regional heterogeneity	Territorial extension	Persistent outbreaks

**Table 4:** Proposed priorities for strengthening invasive *Aedes* mosquito surveillance and arbovirus preparedness in Spain.

**Area**	**Current Challenge**	**Proposed Action**
Entomological surveillance	Uneven surveillance intensity across autonomous communities	Harmonise surveillance protocols and expand monitoring in under-sampled regions
Citizen science	Limited integration of community-generated data	Strengthen integration of Mosquito Alert into routine surveillance
Digital surveillance	Delayed detection of emerging trends	Incorporate digital epidemiology and real-time reporting systems
Artificial intelligence	Limited predictive capacity	Develop AI-based ecological suitability and outbreak prediction models
Inter-institutional coordination	Regional heterogeneity	Improve data sharing between autonomous communities and national authorities
Public awareness	Variable community participation	Expand education and risk communication campaigns

## Data Availability

No new data were created or analyzed in this study. Data sharing is not applicable to this article.
